# SUMO2 rescues neuronal and glial cells from the toxicity of P301L Tau mutant

**DOI:** 10.3389/fncel.2024.1437995

**Published:** 2024-12-12

**Authors:** Franca Orsini, Rosaria Pascente, Annacarla Martucci, Sara Palacino, Paul Fraser, Ottavio Arancio, Luana Fioriti

**Affiliations:** ^1^Department of Neuroscience, Istituto di Ricerche Farmacologiche Mario Negri IRCCS, Milan, Italy; ^2^Tanz Centre for Research in Neurodegenerative Diseases, University of Toronto, Toronto, ON, Canada; ^3^Department of Medical Biophysics, University of Toronto, Toronto, ON, Canada; ^4^Department of Pathology and Cell Biology, Taub Institute for Research of Alzheimer’s Disease and the Aging Brain, Columbia University, New York, NY, United States

**Keywords:** Tau, neuron, astrocytes, microglia, SUMO, AAV6

## Abstract

**Introduction:**

Abnormal intracellular accumulation of Tau aggregates is a hallmark of Alzheimer’s disease (AD) and other Tauopathies, such as Frontotemporal dementia (FTD). Tau deposits primarily affect neurons, but evidence indicates that glial cells may also be affected and contribute distinctively to disease progression. Cells can respond to toxic insults by orchestrating global changes in posttranslational modifications of their proteome. Previous studies suggest that SUMOylation, a posttranslational modification consisting of conjugation of SUMO (Small ubiquitin-like modifier) to target proteins, was decreased in the hippocampus of AD patients and in animal model of AD compared with controls. This decrease in SUMOylation was correlated with increased Tau pathology and cognitive decline. Other studies have reported increased levels of SUMO in AD brains. The goal of our study was to evaluate whether SUMO conjugation modifies the neurodegenerative disease pathology associated with the aggregation-prone mutant TauP301L, in neurons and in glial cells.

**Methods:**

We used viral approaches to express mutant TauP301L and SUMO2 in the hippocampus of wild-type mice. We assessed Tau distribution by immunostaining and Tau aggregation by insolubility assays followed by western blotting. We assessed neuronal toxicity and performed cell count and shape descriptor analyses on astrocytes and microglial cells.

**Results:**

We found that mutant TauP301L, when expressed exclusively in neurons, is toxic not only to neurons but also to glial cells, and that SUMO2 counteracts TauP301L toxicity in neurons as well as in glia.

**Discussion:**

Our results uncover an endogenous neuroprotective mechanism, whereby SUMO2 conjugation reduces Tau neuropathology and protects against toxic effects of Tau in glial cells.

## 1 Introduction

SUMOylation is a process in which SUMO (Small ubiquitin-like modifier) proteins are covalently attached to specific lysine residues in target proteins, thereby regulating various aspects of protein function (Bouchard et al., 2021; Vertegaal, 2022), including their stability, subcellular localization, and interaction with binding partners (Celen and Sahin, 2020). SUMOylation is implicated in several physiological (Mendler et al., 2016) and pathological conditions, such as cancer (Yang et al., 2018), stroke (Yang et al., 2008; Bernstock et al., 2018) and more recently has emerged as a critical player in neurodegenerative diseases (Anderson et al., 2017; Maruyama et al., 2018; Ford et al., 2020). To date, four SUMO isoforms (SUMO1 to SUMO4) have been isolated in mammalian cells with SUMO1-3 being expressed in neurons (Suk et al., 2023), and SUMO1 being the most studied by far (Cho et al., 2015, p. 1). At the amino acid level, SUMO1 is about 50% identical to SUMO2, while SUMO2/3 show a high degree of similarity to each other and are distinct from SUMO1 (Wang and Matunis, 2024).

In Alzheimer’s disease (AD) models, alterations in SUMOylation, including changes in the levels of SUMO1 (Knock et al., 2018; Maruyama et al., 2018) or SUMO2/3 isoforms have been reported (Lee L. et al., 2014). However, the specific role and levels of SUMO2 in AD pathogenesis remain less well-defined compared to SUMO1 (Matsuzaki et al., 2015). Some studies have suggested that SUMOylation levels, including SUMO2, may be dysregulated in AD brains (McMillan et al., 2011; Lee L. et al., 2014; Ficulle et al., 2018). For example, one study found decreased SUMOylation levels, including SUMO2/3 conjugates, in the hippocampus of AD patients and in animal model of AD (Lee L. et al., 2014) compared with controls. This decrease in SUMOylation was correlated with cognitive decline. In contrast, other studies have reported increased levels of SUMO2 in AD brains (Patel et al., 2019). The discrepancies in these findings may reflect differences in the experimental methodologies, brain regions studied, or disease stages analyzed. Additionally, the complex and context-dependent nature of SUMOylation (Sang et al., 2011) in cellular processes makes it challenging to elucidate its precise role in AD pathogenesis.

SUMO2 conjugation has been associated with the response to conditions of stress (Enserink, 2015; Manza et al., 2004; Sang et al., 2011), where changes in global SUMO2ylation are associated with improved outcomes (Lee et al., 2006; Lee et al., 2011; Lee Y.-J. et al., 2014). In this context, an increase in SUMO2 conjugation might occur at early stages of AD, when amyloid-beta (Hardy and Higgins, 1992) and Tau oligomers first start to accumulate in the brain (Braak and Braak, 1995). Under these circumstances, SUMO2 conjugation may be activated to respond to the stress conditions caused by amyloid-beta and Tau oligomers, in an attempt to counteract their toxicity. At later time points, however, when their toxicity prevails and the pathology worsen, SUMO2 conjugation might be reduced.

Transgenic mice expressing mutant forms of human Tau protein, such as P301L or P301S mutations, develop Tau pathology, including Tau hyperphosphorylation, aggregation, and neurofibrillary tangle formation (Yoshiyama et al., 2007; Lewis et al., 2000), similar to what is observed in human Tauopathies (Kovacs, 2017). These models have been extensively used for studying disease mechanisms and testing potential therapies, however, while changes in SUMO conjugation have been explored in models of amyloid beta deposition (Lee L. et al., 2014; Nisticò et al., 2014), the effects of overexpression of mutant forms of Tau on SUMO conjugation have not been investigated so far.

To test the hypothesis that changes in SUMO2 occur early and to understand their contribution to disease progression, we developed a model of transition from acute to chronic Tau toxicity by injecting TauP301L AAVs into the hippocampus of wild type animals. The goal of our study was to evaluate whether changes in SUMO2 conjugation modifies the neurodegenerative disease pathology associated with the aggregation-prone mutant TauP301L, in neurons and glial cells.

We expressed a mutant form of Tau, TauP301L, which is associated with frontotemporal dementia, (FTD) for 17 weeks, and found that TauP301L induces an increase in SUMO2 conjugation. To determine whether the increase in SUMO2 was beneficial or detrimental, we co-infected a group of mice with both TauP301L and SUMO2 AVVs to further increase SUMO2 conjugation. We reasoned that if the increase in SUMO2 was indeed a protective response to cellular stress triggered by Tau toxicity, a further increase would be beneficial. Indeed, we found that co-infection with SUMO2 prevented neuronal loss induced by the expression of TauP301L. Moreover, we found that neuronal expression of TauP301L induced toxicity not only in neurons but also in astrocytes and microglial cells, and SUMO2 also rescued astrocytes and microglial cells from Tau toxicity.

## 2 Methods

### 2.1 Animals and adeno-associated viral transduction

Procedures involving animals and their care were conducted in conformity with institutional guidelines in compliance with national and international laws and policies. Homozygous JNPL3 mice were purchased from Taconic (USA), strain ID #2508. 9–10 weeks old C57Bl/6 J male mice (Envigo, Italy) were anesthetized with 3% isoflurane in N2O/O2 (70/30%) and maintained by 1.5 to 2% isoflurane in the same gas mixture. Stereotactic intrahippocampal injections of 1.3*10^9^ infective units (IU) of AAV1 TauP301L alone or in combination with the same units of AAV1 SUMO2 (Vector biolabs) were performed at the following coordinates: posterior -1.94mm, lateral +1.4 and −1.4 mm, ventral −1.8 and −1.6 mm. The AAV constructs used were the following: AAV1-CMV-eGFP-P2A-Tau-2N4R(P301L)-HA, AAV1-CMV-RFP-CMV-hSUMO2. Four months after AAV injections mice were deeply anesthetized. For histology mice were perfused with 20 mL of ice-cold phosphate-buffered saline (PBS) 0.1 mol L-1, pH 7.4 followed by 50 mL of chilled paraformaldehyde (4%, PFA) in PBS. The brains were removed from the skull and post-fixed overnight at 4°C, then dehydrated with 30% sucrose (Sigma-Aldrich) in 0.1 mol L-1 PBS for 24 h at 4°C, then frozen in n-pentane for 3 min at −45°C and stored at −80°C until use. For biochemical analysis mice were perfused with 20 mL of ice-cold PBS, the brains were removed from the skull, the hippocampi were dissected and snap frozen.

### 2.2 Protein extraction and western blot

Hippocampi were dissected from the brain and washed in ice-cold PBS. The tissues were homogenized in extraction buffer (50 mM Tris–HCl [pH7.5], 50 mM KCl, and 10 mM MgCl2) supplemented with complete protease and phosphatase inhibitor cocktails (Roche Applied Science) and N-Ethylmaleimide (NEM) 20 mM. Cell debris were removed by centrifugation at 7000 rpm for 10 min. Twenty ug of total proteins were run into 4–20% gradient tris-glycine TGX™ Precast Protein Gels (Bio-Rad) and transferred onto nitrocellulose membranes using semi-dry Trans-Blot® Turbo™ Transfer System (Bio-Rad). Membranes were blocked in 3% BSA for 1 h RT and then probed with mouse anti-HA antibody (1:1000, # MMS-101R, COVANCE), chicken anti-total Tau (1:5000, PA595648, Invitrogen), mouse anti-vinculin (1:5000, # V9264, Sigma), rabbit and rat anti-SUMO2 (1:1000, # 3742, Abcam and 1:1000, #MABS1139, Sigma, respectively) overnight at 4°C. Anti-mouse HRP (1:10000, #115-035-174, Jackson Immunoresearch), anti-rabbit HRP (1:10000, NA934, GE Healthcare), anti-rat (1:5000, #98164, Cell Signaling) and anti-chicken Dylight 488 (1:3000, #SA5-10070, Thermo) were used as secondary antibodies. Western blot images were acquired with ChemiDoc MP Imaging System (Bio-Rad).

### 2.3 Detergent insolubility assay

One hundred μg of hippocampi homogenates (as described above) were incubated with 0.5% Triton X-100; 0.5% NP-40; 0.5% sodium deoxycholate in 50 mM Tris–HCl (pH 7.5) for 30 min on ice and centrifuged in a TLA 55 rotor at 65,000 rpm for 40 min at 4°C (Drisaldi et al., 2015). Soluble (supernatant) and insoluble (pellet) fractions were analyzed by western blotting.

### 2.4 Semi-denaturating detergent agarose gel electrophoresis (SDD-AGE)

Protein homogenates from transduced hippocampi (40 μg) were incubated in the sample buffer 2X (25 mM Tris, glycine [pH 8.3], 0.04% SDS, 5% glycerol, and 0.025% bromophenol blue) at room temperature (∼25°C) for 7 min and then loaded into 1.5% low gelling agarose (A3414, Sigma) gel (Drisaldi et al., 2015) containing 0.02% SDS. Proteins were transferred onto a nitrocellulose membrane and immunoblotted with mouse anti-HA antibody followed by anti-mouse HRP conjugated secondary antibody.

### 2.5 Histology

Serial coronal brain sections (20 μm thick) were cut on a cryostat for histological analysis.

### 2.6 Nissl staining

Nissl staining was carried out to examine the toxicity of the transduced TauP301L on CA1 and CA3 hippocampal neurons. Briefly, the brain sections were dehydrated through graded alcohols (70, 96, 100%) followed by xylene and then rehydrated. After the rehydration, the slices were stained with a solution of 0.5% cresyl violet acetate (# C5042, Sigma) and the staining was further differentiated by washing in solutions with increasing concentration of alcohol (70, 96, 96 + 3% CH3COOH, 100%) then placed in xylene and covered using DPX mounting agent (Orsini et al., 2012).

### 2.7 Immunohistochemistry

Twenty microns thick coronal section were treated with H_2_O_2_ 1% for 10 min to inactivate endogenous peroxidase activity. Blocking solutions and incubation with primary antibodies were as follow: GFAP: blocking in 10% fetal bovine serum (FBS) and 0.5% Triton in PBS for 1 h, followed by overnight incubation at 4°C with the primary antibody mouse anti-GFAP in blocking solution (1:2000; Chemicon). Iba1: blocking solution included 10% normal goat serum (NGS) and 0.3% Triton in PBS for 1 h, followed by overnight incubation at 4°C with primary antibody rabbit anti-Iba1 (1:200; Wako) in a solution containing 0.2% Triton and 2% NGS in PBS. The day after, slices were washed with PBS and incubated with biotinylated anti-mouse and anti-rabbit secondary antibodies (for GFAP and Iba1 respectively) for 1 h at RT, followed by incubation with the Vectastain Elite ABC reagent (#PK-6100, Vector Laboratories). For each reaction, immunoreactivity was detected using 3,3′-diaminobenzidine (# K3468, Dako).

### 2.8 Quantitative analyses of IHC sections

For quantitative analyses, brain sections were acquired at ×20 by Olympus BX-61-*VS* microscope, interfaced with *VS*-ASW-FL software (Olympus, Germany). Acquisition was done over 10-μm thick stacks, with a step size of 2 μm. The different focal planes were merged into a single stack by mean intensity projection to ensure consistent focus throughout the sample. For Nissl staining two coronal sections at AP −1.5 mm and −2.0 mm from bregma were acquired and analyzed using Fiji software. CA1 thickness was calculated by superimposing a grid on the acquired image and by drawing 11 and 17 lines (for −1.5 mm and −2.0 mm APs respectively) over the pyramidal layer. CA3 neuronal density was measured by drawing a Region of Interest (ROI) over the CA3 region and by counting the positive pixels/total assessed pixels, reported as the percentage of total stained area. For GFAP and Iba1 staining three coronal sections at AP −1.35, −1.75 and −2.15 from bregma were acquired and the entire hippocampus was analyzed by Fiji software as previously described (Orsini et al., 2016) in order to outline glial cells.

### 2.9 Immunofluorescence

For AAVs distributions and Tau expression, brain slices were rinsed in PBS, blocked and permeabilized with 5% normal goat serum (NGS) + 0.3% Triton for 1 h at RT and then incubated with rabbit anti-HA 1:500 antibody (#9110, Abcam) diluted in Triton 0.1% + NGS 1% overnight at 4°C. After 3 rinses the slices were incubated with anti-rabbit Alexa647 antibody (1:500, Thermo) for 1 h RT, rinsed and covered with ProLong™ Gold Antifade Mountant (P36930, Invitrogen) and coverslip. For GFAP and Iba1, sections were processed as described in immunohistochemistry section with the exception of secondary antibodies. Anti-mouse and anti-rabbit Alexa647 conjugated antibodies (1:500, Thermo) were used.

### 2.10 Confocal acquisition

The AAVs distributions were evaluated by confocal analysis. Hippocampi were acquired by stitching adjacent 20X frames using a scanning sequential mode to avoid bleed-through effects by Confocal A1 system (Nikon), equipped with confocal scan unit with 405 nm, 488 nm, 561 nm, 640 nm laser lines. Three-dimensional and higher magnification images were acquired using a 60X water immersion objective over a 8- to 10-μm *z* axis with a 0.273-μm step size and processed by using Imaris software (Bitplane).

### 2.11 Statistical analysis

Data are expressed as mean ± standard error of the mean (SEM) and graphed as scatter plots. GraphPad Prism 9 software was used for statistical analysis. All data were tested for normal distribution using the Shapiro–Wilk normality test. For >2 groups, results were analyzed by one-way ANOVA followed by Tukey’s multiple comparison test. In the case of =2 groups, data were compared using the unpaired t-test if they were normally distributed or the Mann–Whitney test if they were not. Additional information on the tests used is provided in the figure legends.

## 3 Results

### 3.1 Overexpression of hTauP301L in the hippocampus of JNPL3 mice induces a reduction of SUMO2 conjugation

We recently reported that SUMO2 conjugation is reduced in the hippocampus of PS19 mice, which express human Tau with the P301S mutation (Yoshiyama et al., 2007), at 8 months of age, when Tau pathology and the associated cognitive impairments and neurodegeneration are already present (Orsini et al., 2022).

**Figure 1:**
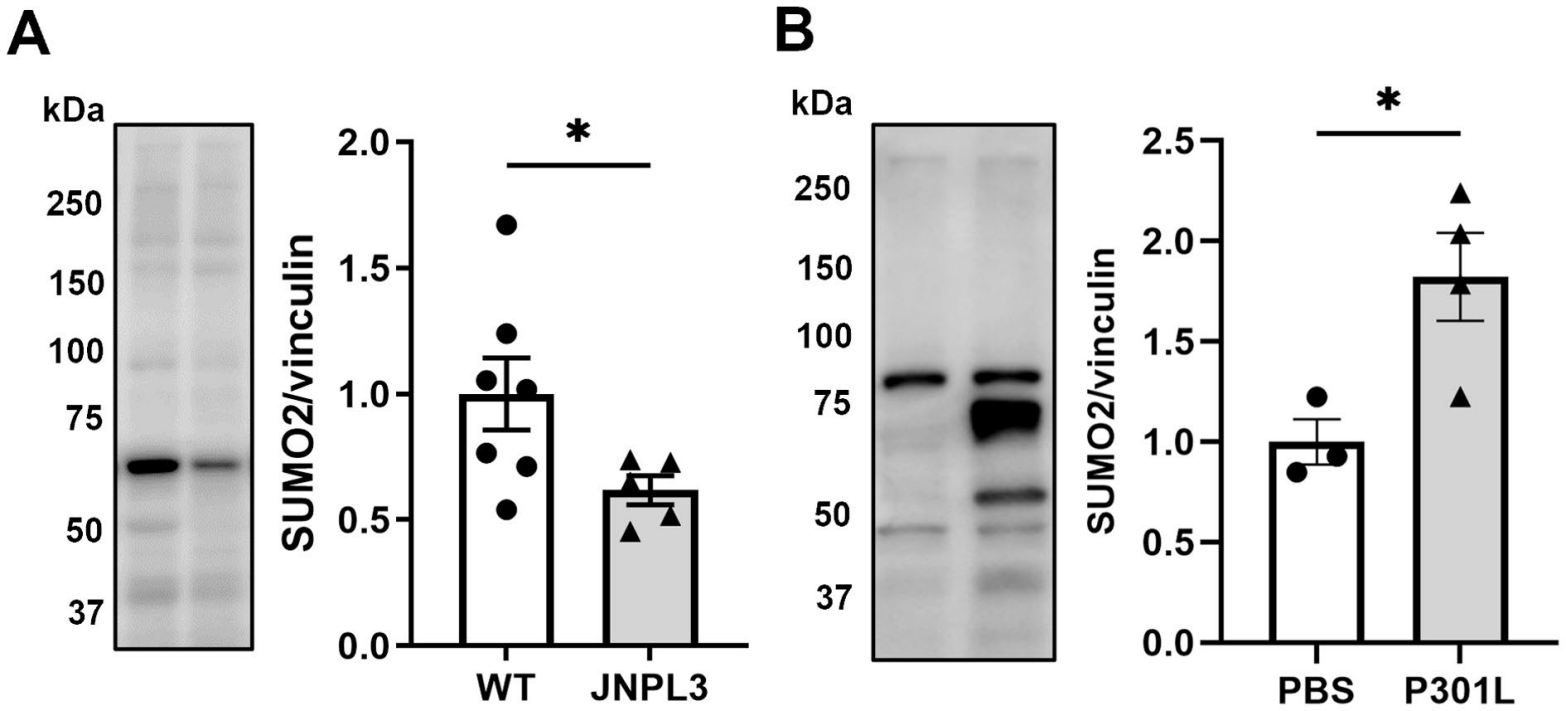
SUMO2-conjugation in models of mutant Tau overexpression. **(A)** Representative image and quantification of SUMO2-conjugation in the brains of wild-type (WT) and human TauP301L overexpressing (JNPL3) mice at 5 months of age by western blotting (WB). Chronic expression of human TauP301L leads to a significant reduction of SUMO2 conjugation in JNPL3 compared to WT mice (*n* = 7–5). Since data were not normally distributed (by Shapiro–Wilk normality test) Mann Whitney test was applied, *p < 0.05. **(B)** Analysis of SUMO2-conjugation in the hippocampi of 11 weeks-old WT mice injected with PBS or AAV transducing TauP301L (1.3×10^9^ IU) for 17 weeks. The quantification showed that the sub-chronic expression of TauP301L induced an increase in the SUMO2 conjugation compared to PBS, *n* = 3–4, unpaired t-test, *p* < 0.05.

Here we tested whether similar changes are present in the hippocampus of JNPL3 mice (Lewis et al., 2000), which express human Tau with the P301L mutation, at 5 months, a time point when there are only modest changes of Tau hyperphosphorylation and aggregation in the hippocampus (Lewis et al., 2000). We performed western blotting analyses on hippocampal extracts from JNPL3 and wild type mice (sex mixed cohort for both genotypes) and compared the extent of SUMO2 conjugation. We found a significant 50% reduction in SUMO2 conjugation in JNPL3 mice compared to aged-matched wild-type (Figure 1A).

### 3.2 Expression of hTauP301L by AAVs in mouse hippocampus induces an increase of SUMO2 conjugation

JNPL3 mice express significantly higher amount of h Tau compared to endogenous levels (Lewis et al., 2000). Moreover, they express h Tau under the PrP promoter, starting from late embryonal development. To avoid any confounding effect due to the high levels of overexpression of mutant Tau, and to further reduce confounding effects due to expression during development, we utilized a viral approach to express mutant Tau in a temporal and spatially restricted way by means of infecting adult wild-type mice with AAV particles of hTauP301L with an HA tag. We opted for a mild overexpression of hTauP301L-HA only in the dorsal hippocampus. This was achieved by injecting 1.3E^9^ infective units (IU) into the hippocampus of wild-type mice and by expressing them for 17 weeks. At the end of the 17 weeks, hippocampi were collected and analyzed by western blot to measure the expression levels of the transduced Tau, compared to the endogenous protein. We found that the hTauP301L protein was only 10% of the total Tau expressed by the infected mice (Supplementary Figure 1). Under these experimental conditions we observed 80% increase in SUMO2 conjugation compared with PBS injected mice, suggesting that time of expression and amount of mutant Tau expressed could influence the process of SUMO2 conjugation (Figure 1B).

### 3.3 Overexpression of SUMO2 reduces the formation of hTauP301L insoluble aggregate

**Figure 2:**
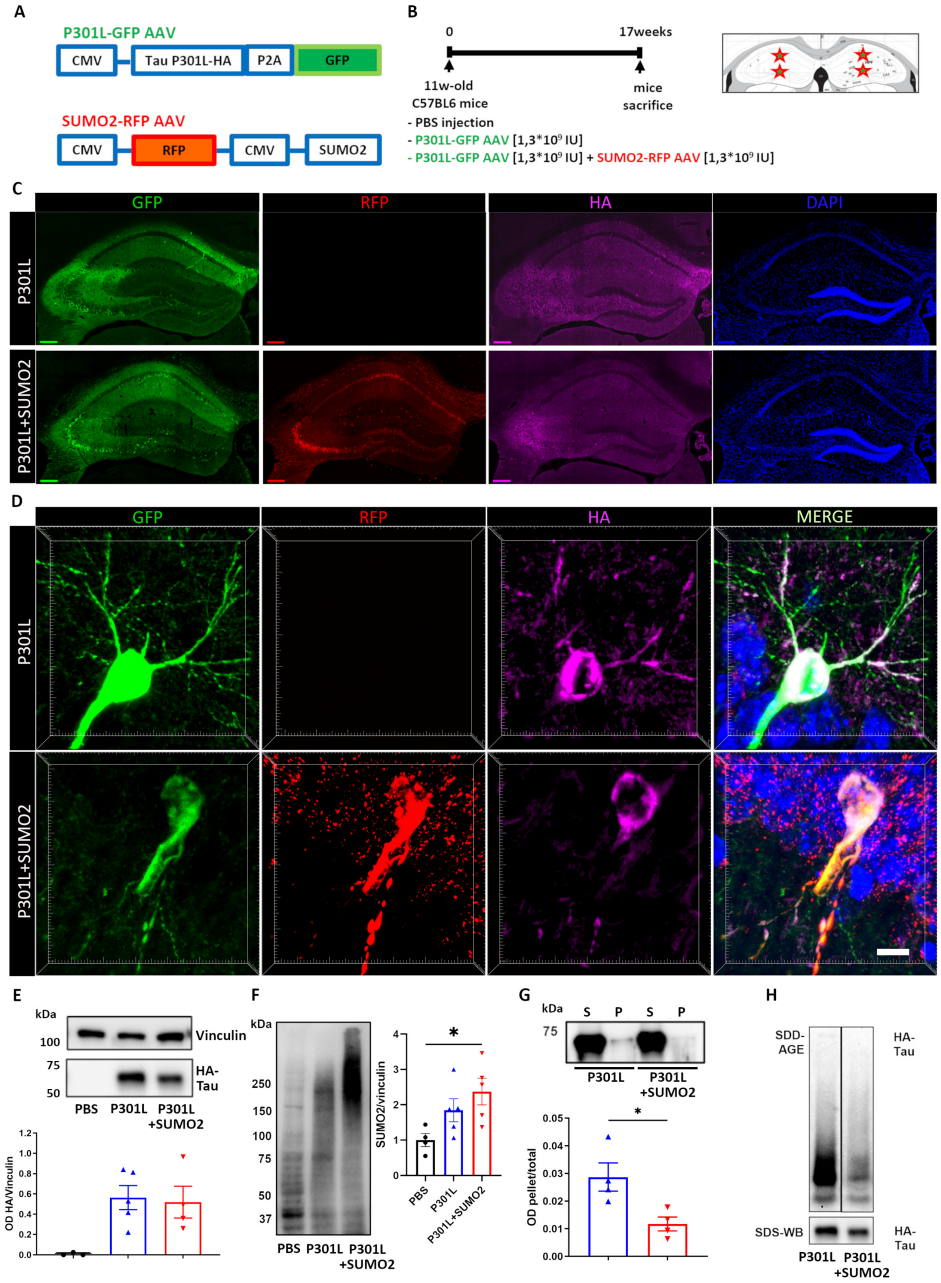
AAV model of hTauP301L expression in mice. **(A)** Eleven weeks old wild-type mice received hippocampal injections of PBS, AAV transducing 2N4R hTauP301L (1.3×10^9^ IU) or AAV transducing 2N4R TauP301L (1,3×10^9^ IU) together with AAV transducing SUMO2 (1,3×10^9^ IU). TauP301L included an HA tag at the C terminus of Tau and was expressed together with GFP under the same promoter. SUMO2 was expressed together with RFP, under a different promoter. **(B)** Schematic representation of the experimental design showing the injection sites and time of expression. **(C)** Representative images of transduced hippocampal slices: AAV TauP301L distribution in green, AAV SUMO2 distribution in red, HA immunostaining in magenta shows the TauP301L protein expression and nuclei (stained with DAPI) in blue, scale bar 200um. **(D)** Representative magnifications showing the soma and principal arborizations of one neuron in the CA1 transduced with TauP301L (upper panels) or TauP301L in combination with SUMO2 (lower panels). The two panels on the right are the merge of the single channels (scale bar 10 μm). **(E)** Quantification of HA immunoblot demonstrates equal expression of TauP301L protein in both AAV TauP301L and AAV TauP301L+ AAV SUMO2 groups. **(F)** Western blot and quantification confirm that SUMO2 conjugation is increased by transient expression of TauP301L and it is further increased by AAV SUMO2 injection in transduced hippocampi. One-way ANOVA followed by Tukey’s test, *p < 0.05 **(G)** Detergent insolubility assay and **(H)** SDD-AGE demonstrate that aggregation of transduced exogenous Tau is lower in TauP301L − + AAV SUMO2 than in TauP301L alone. Un-paired t-test, *p < 0.05.

To determine whether the increase of SUMO2 conjugation induced by expression of hTauP301L is beneficial or detrimental, we co-infected a group of mice with both hTauP301L and SUMO2 AVVs (Figure 2A), to further increase SUMO2 conjugation in the hippocampus, for 17 weeks (Figure 2B). We found in both AAV groups a robust expression of human Tau protein checked by HA tag immunofluorescence and detected its hippocampal distribution, together with that of GFP and RFP, used as reporters for TauP301L-HA and SUMO2 AAVs, respectively (Figure 2C). Despite we used the universal CMV promoter to drive expression of TauP301L-HA, we found that Tau signal was restricted to neurons (Figure 2D).

It has been previously reported that overexpression of Tau in the hippocampus is accompanied by hyperphosphorylation and aggregation of Tau. Thus, we tested whether increase of SUMO2 could affect these phenomena. First, we assessed whether overexpression of SUMO2 had any effect on the stability of Tau. Western blotting analyses established that SUMO2 overexpression did not affect the amount of TauP301L expressed in transduced hippocampi (Figure 2E). As a control, we also confirmed that transduction of SUMO2 AAVs induced a significant increase of SUMO2 conjugation (200% increase vs. PBS injected mice, Figure 2F). Next, we performed detergent insolubility assay and semi-denaturing agarose gel electrophoresis (SDD-AGE) to assess the aggregation state of hTauP301L when expressed alone or in combination with SUMO2. We found that hTauP301L was modestly insoluble to non-ionic detergents and that SUMO2 significantly reduced the degree of insolubility of hTauP301L (60% reduction vs. TauP301L alone, Figure 2G). By SDD-AGE analyses, we also found that hTauP301L formed SDS-resistant oligomers, that were reduced by overexpression of SUMO2 (Figure 2H). Finally, we assessed the phosphorylation of several residues: Ser202-Thr205, Thr181, Thr212-Ser214, Ser396 and Ser404. We found a significant change of pTau Ser202-Thr205, Ser396 and Ser404 in hippocampal extracts of mice expressing TauP301L (Supplementary Figure 2). The co-expression of SUMO2 reduced significantly the pTau Ser396 and Ser404 hyperphosphorylation, bringing it back to control levels, while Ser202-Thr205 hyperphosphorylation was only partially reduced, but remained higher than the control group (Supplementary Figure 2).

### 3.4 Expression of hTauP301L induces neuronal loss which is rescued by SUMO2 overexpression

**Figure 3:**
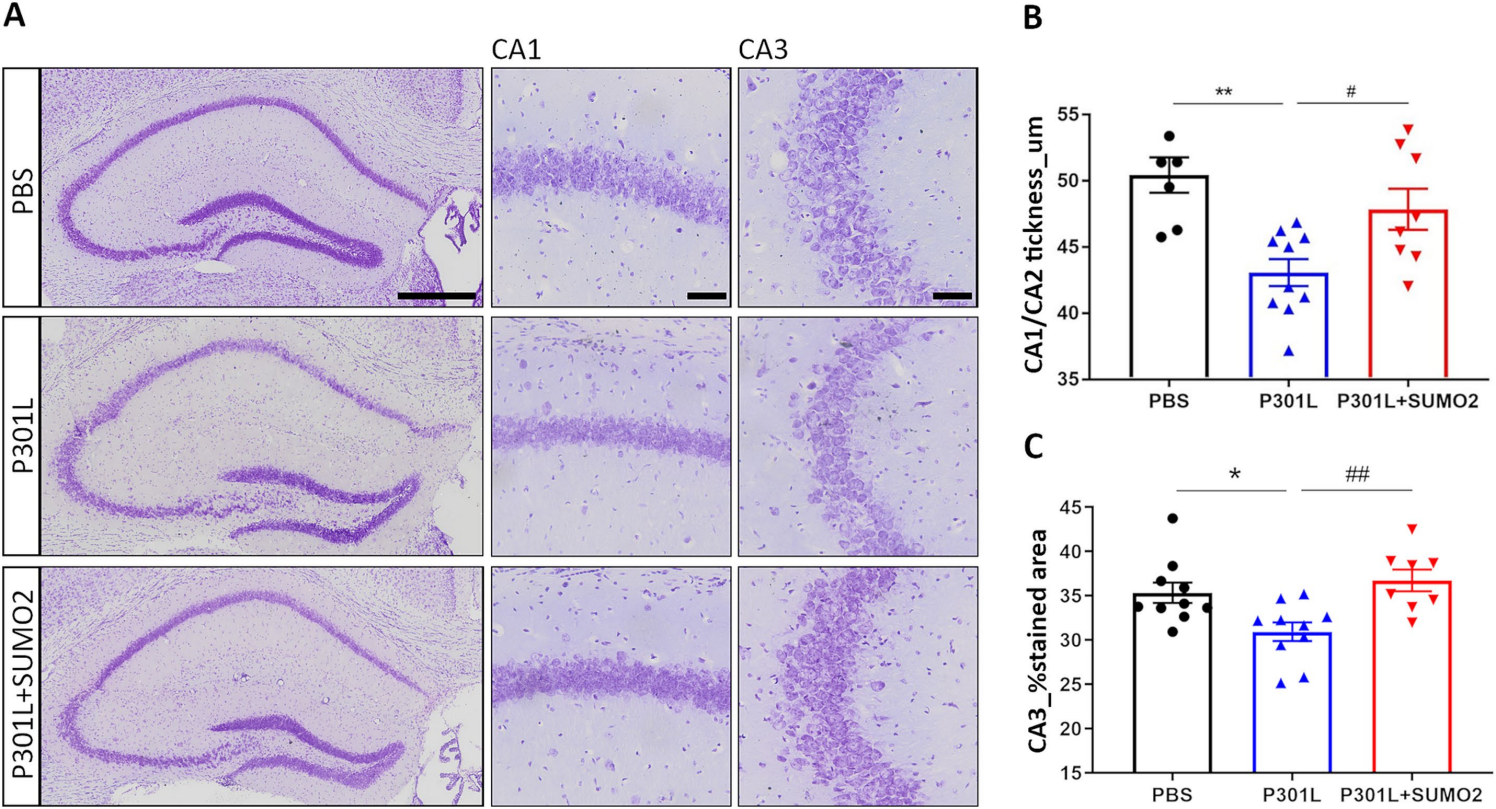
Analysis of hippocampal CA1 and CA3 thickness in AAV transduced mice. **(A)** Representative images of hippocampi stained with Cresyl violet showing the entire hippocampus (left panel), CA1 (middle panel) and CA3 (right panel) magnifications (scale bars 50 μm) of mice injected with PBS, P301L-GFP AAV alone, or P301L-GFP AAV in combination with SUMO2-RFP AAV. Scale bar 500 μm. **(B)** Quantification of the CA1/CA2 thickness and **(C)** the % stained CA3 area demonstrates a reduction of both parameters in animals expressing TauP301L, and a rescue in mice transduced with P301L + SUMO2 AAVs compared with P301L AAV alone; one-way ANOVA followed by Tukey’s multiple comparisons test; #*p* < 0,05, **p < 0,001 (*n* = 6, 10 and 8) and *p < 0.05, ##*p* < 0.001 (*n* = 10, 10 and 8) respectively.

Overexpression of Tau in the hippocampus, either wild-type or mutant TauP301L, has been reported to provoke dramatic degeneration of pyramidal neurons in CA1/2 within weeks (Jaworski et al., 2009). Thus, we tested whether neuronal loss also occurred in our experimental conditions of much lower expression of mutant hTauP301L. We performed Cresyl Violet staining of hippocampi of mice injected with PBS, P301L-GFP AAV alone or in combination with SUMO2-RFP AAV and analyzed CA1/2 and CA3 area (Figure 3A). Quantification of the CA1/CA2 thickness (Figure 3B) and the % stained CA3 area (Figure 3C) demonstrates a reduction of both parameters in TauP301L expressing animals, compared to PBS injected mice (20 and 10% reduction, respectively). Next, we tested whether increase of SUMO2 could be beneficial to counteract the toxicity of Tau. We found that SUMO2 overexpression rescued the neuronal loss observed both in CA1/2 (Figure 3B) and CA3 regions (Figure 3C). We also observed the presence of chromatolysis (Kusaka et al., 1988; Levine et al., 2004; McIlwain and Hoke, 2005) in neurons expressing high levels of TauP301L. These neurons were not lost yet, but they clearly showed signs of degeneration, as demonstrated by the presence of degenerating nuclei and axons (Supplementary Figure 3).

### 3.5 Expression of TauP301L in neurons induces atrophy of GFAP-positive astrocytes in the hippocampus and is rescued by SUMO2

The toxic effects of abnormal Tau aggregates have been studied mostly in neurons, however emerging evidence suggests that Tau pathology can also impact glial cells, particularly astrocytes and microglia. Tau deposits in astrocytes are frequently found in AD and other Tauopathies (Hallmann et al., 2017; Koga et al., 2014; Reid et al., 2020). However, since astrocytes do not express Tau, the inclusions have been suggested to be of neuronal origin.

**Figure 4:**
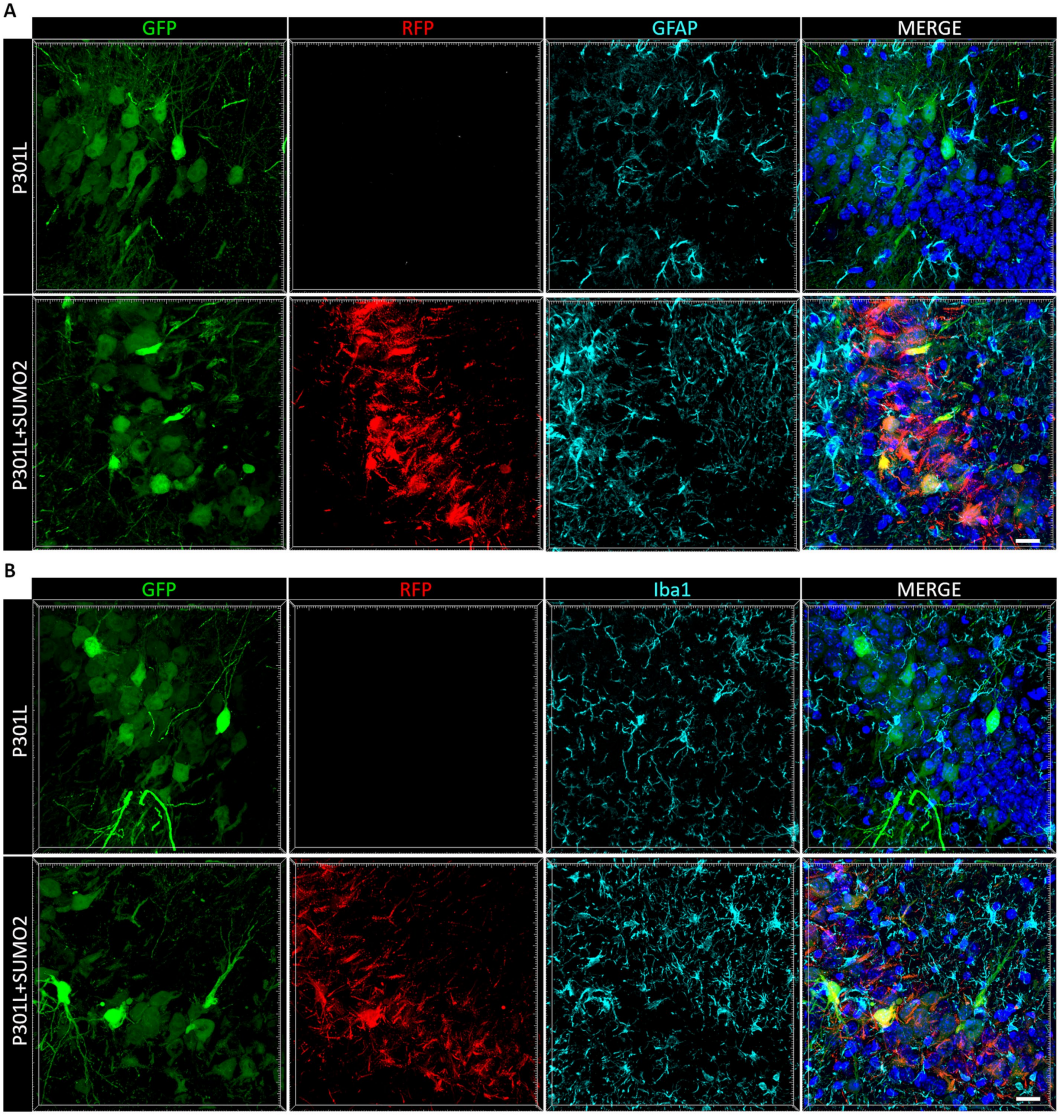
Analysis of AAVs transduction in non-neuronal cells. Representative images of hippocampal sections of mice transduced with AAV TauP301L (green) alone or in combination with AAV SUMO2 (red) stained with a marker of astrocytes (GFAP, in **A**) and **(B)** a marker of microglial cells (Iba1) shown in cyan. Nuclei stained with DAPI (blue) are shown in the right panels merged with the other channels. Neither TauP301L nor SUMO2 AAV are expressed in GFAP-or Iba1-positive cells, scale bar 20 μm.

**Figure 5:**
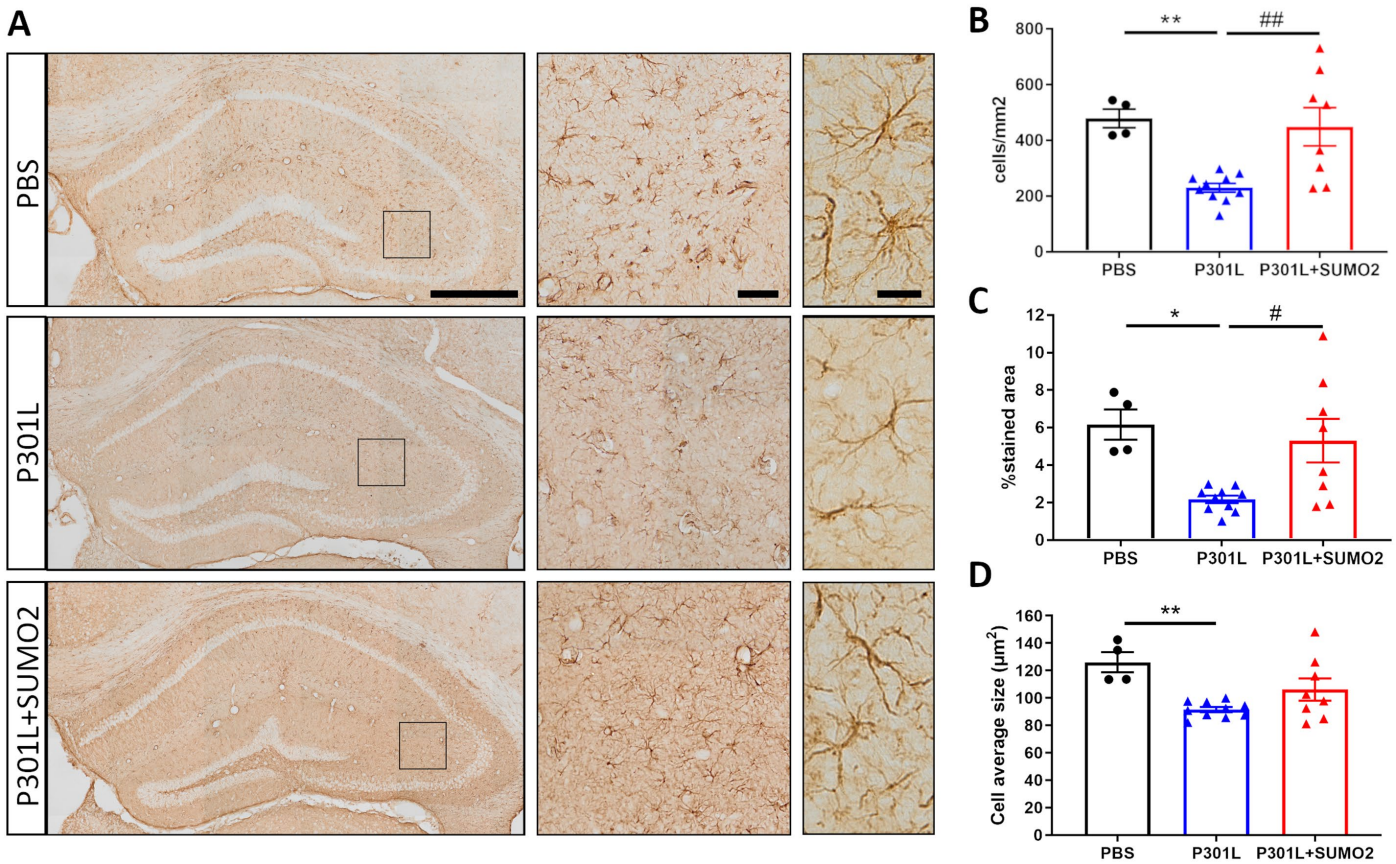
Morphometric analyses of GFAP positive astrocytes in the hippocampi of AAV transduced mice. **(A)** Representative GFAP images of hippocampal slices of mice injected with PBS, P301L-GFP AAV alone, or P301L-GFP AAV in combination with SUMO2-RFP AAV stained with GFAP (scale bar 500 μm), 40x magnification (middle panels, scale bar 50 μm) corresponding to the squares and 60x magnification highlighting 2–3 cells in the brain tissue (right panels, scale bar 20 μm). The quantification of the number of GFAP+ cells/mm2 **(B)**, the percentage of GFAP stained area **(C)**, and the astrocyte average size **(D)** reveal that Tau expression induces an atrophy of astrocytes that is prevented by SUMO2 co-transduction; one-way ANOVA followed by Tukey’s multiple comparisons test; * vs. CTR or # vs. P301L + SUMO2, # or *p < 0.05, ## or **p < 0.001, *n* = 4, 10 and 8.

When we looked at Tau expression, we observed that the HA signal were mostly restricted to neurons (Figures 2C,D), even if we used the universal CMV promoter to drive expression of TauP301L-HA. To exclude that Tau was expressed also by astrocytes and microglia we performed co-staining of HA with GFAP (Figure 4A) or Iba1 (Figure 4B). We did not observe any colocalization between HA and glial markers demonstrating that both hTauP301L and SUMO2 were not expressed in astrocytes or microglia cells. Next, we conducted a detailed morphometric analysis in astrocytes of mice injected with hTauP301L AAV (Figure 5A), which revealed 50% reduction of the number of astrocytes (Figure 5B), 60% decrease of total surface area (Figure 5C), and 20% reduction of the cells average size (Figure 5D), compared with control hippocampi. Our results suggest that astrocytes cells are not hypertrophic, instead present an atrophic morphology. Moreover, our data suggest that TauP301L expression in neurons induces astrocytes degeneration and loss. We also found that co-expression of SUMO2 rescued the cell loss (Figure 5B) and amount of total GFAP signal (Figure 5C), while did not affect the size of astrocytes. Overall these findings indicate that TauP301L expressed in neurons also produces a toxic effect on astrocytes, and that SUMO2 counteracts Tau P301L toxicity toward astrocytes.

### 3.6 Expression of TauP301L in neurons induces atrophy of Iba1-positive microglia in the hippocampus and is rescued by SUMO2

Tau aggregates can activate microglia once released by neurons in the extracellular space (Morales et al., 2013). Activated microglia release cytokines, chemokines, and reactive oxygen species, which further contribute to neuronal damage (Sasaki et al., 2008).

**Figure 6:**
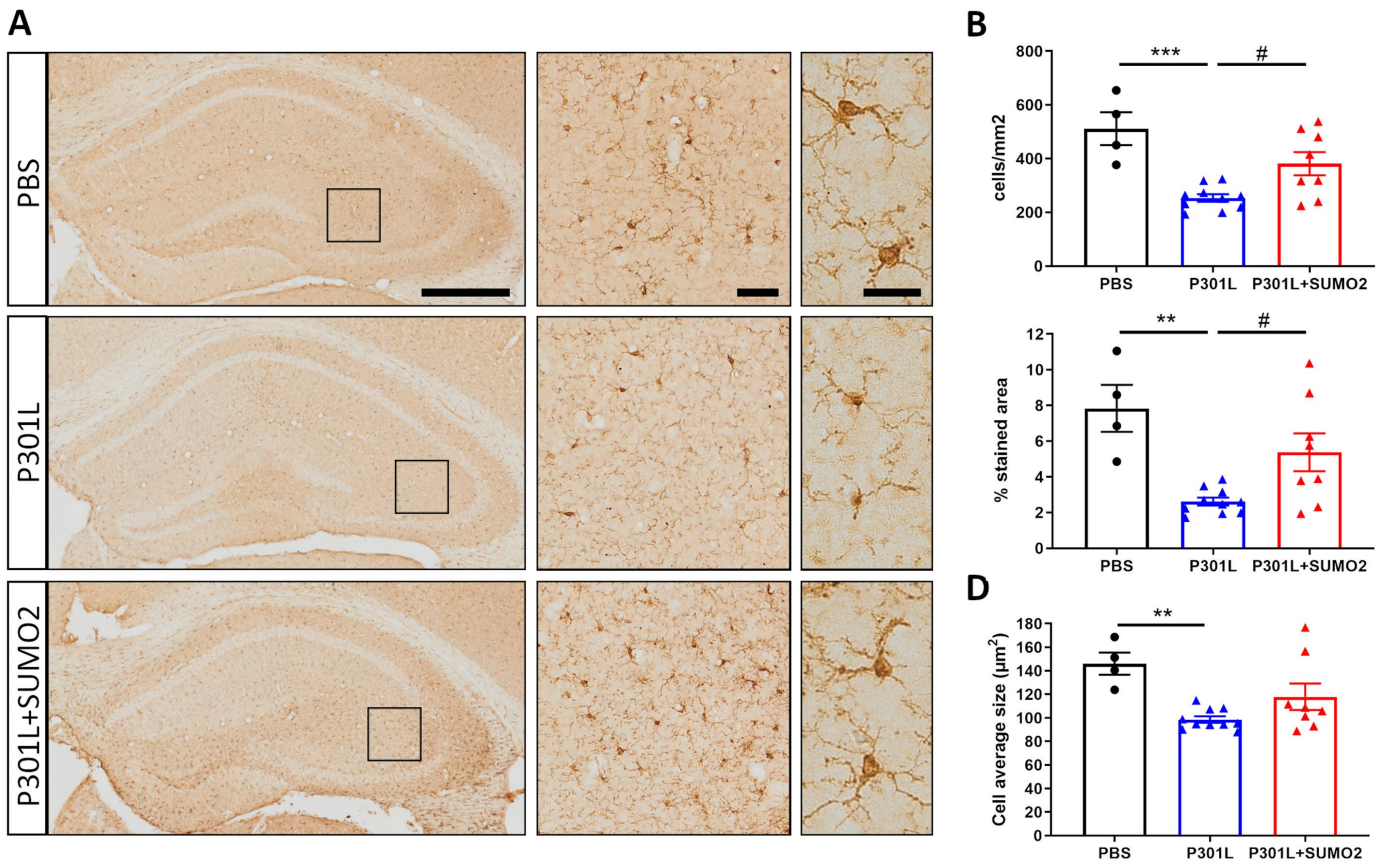
Morphometric analyses of Iba1 positive microglia in the hippocampi of AAV transduced mice. **(A)** Representative images of Iba1 staining of mice injected with PBS, P301L-GFP AAV alone, or P301L-GFP AAV in combination with SUMO2-RFP AAV showing the entire hippocampus (scale bar 500 μm), 40x magnification (middle panels, scale bar 50 μm) corresponding to the squares and 60x magnification highlighting 2–3 cells in the brain tissue (right panels, scale bar 20 μm). The quantification of the number of Iba1+ cells/mm2 **(B)**, the percentage of Iba1 stained area **(C)**, and the microglia average size **(D)** reveal that Tau expression induces a shrinking of microglial cells that is prevented by SUMO2 co-transduction; one-way ANOVA followed by Tukey’s multiple comparisons test; * vs. CTR or # vs. P301L + SUMO2, # or *p < 0.05, ## or **p < 0.001, *n* = 4,10 and 8.

To determine whether neuronal expression of hTauP301L induced microglia activation, we performed detailed morphological analyses of microglial cells stained with Iba1 (Figure 6A). Similar to what we found in astrocytes, our analyses revealed 50% reduction of the number of microglial cells (Figure 6B), 75% decrease of total surface area (Figure 6C), and 30% reduction of the cells average size (Figure 6D), compared with control hippocampi. Our results suggest that microglial cells are not hypertrophic, instead present an atrophic morphology. Moreover, our data suggest that TauP301L expression in neurons induces microglia degeneration and loss. We also found that co-expression of SUMO2 rescued the cell loss (Figure 6B) and amount of total Iba1 signal (Figure 6C), while did not affect the size of microglial cells. Overall these findings indicate that TauP301L expressed in neurons exerts a toxic effect on microglia, and that SUMO2 counteracts these effects.

## 4 Discussion

Changes in SUMO conjugation have been reported in several neurodegenerative disease (Dorval and Fraser, 2007; Yang and Paschen, 2015), including AD, with conflicting evidence pointing to both a reduction (Lee L. et al., 2014; Nisticò et al., 2014) and increase in SUMO conjugation (Chenfei et al., 2022; McMillan et al., 2011). In particular, changes in the neuroprotective SUMO2 isoform and their significance with respect to the progression of the pathology are poorly understood.

In this study, we investigated whether increase of SUMO2 occurs in response to elevation of toxic forms of Tau, and if such increase counteracts or further aggravates Tau toxicity. Previous data demonstrate that increase of SUMO2 conjugation is beneficial during conditions of stress (Ryu et al., 2020; Manza et al., 2004; Enserink, 2015). This led us to reason that an increase of SUMO2 could be beneficial toward Tau-induced toxicity. To test this hypothesis, we designed an experiment in which we overexpressed hTauP301L alone, or hTauP301L and SUMO2, in the hippocampus of adult wild-type mice by AAV, for 4 months. In line with previous data (Jaworski et al., 2009), our AAV model is characterized by accumulation of insoluble forms of hTauP301L and neuronal loss. Moreover, we found that hTauP301L expression in neurons caused toxicity also in astrocytes and microglial cells. Furthermore, we found that increase in SUMO2 significantly reduced the aggregation of hTauP301L, neuronal loss, as well as glial toxicity.

The mechanisms by which SUMO2 can rescue neuronal loss and glial toxicity caused by hTauP301L are currently unknown. Several proteins can be modified by SUMO2 and interestingly Tau itself has been found to be a target of SUMOylation (Dorval and Fraser, 2006). In particular, it was demonstrated that SUMO1 conjugation onto Tau increases Tau phosphorylation and aggregation in transfected HEK cells (Luo et al., 2014), but the consequences of SUMO2 conjugation onto Tau have not been fully investigated. Here we found that overexpression of SUMO2 reduces the aggregation of hTauP301L. However, we did not demonstrate that this effect is mediated by a direct conjugation of SUMO2 onto hTauP301L. This is an important aspect that needs to be further investigated.

How does SUMO2 expressed in neurons prevent hTauP301L toxicity in glial cells? This is an intriguing question that deserves further investigations. One possibility might be that by reducing Tau aggregation in neurons, and by reducing neuronal loss, SUMO2 indirectly protects glial cells because it reduces the release of toxic aggregates of Tau by neurons. In fact, it has been well documented that astrocytes and microglial cells are affected by toxic forms of Tau of neuronal origins (Reid et al., 2020; Hallmann et al., 2017; Nilson et al., 2017). Interestingly the use of viral vectors is particularly useful to address cell specific effects. By selectively expressing mutant Tau in specific cell populations, it is possible to investigate the cell-autonomous effects of Tau pathology on neuronal function and survival or explore the contributions of glial cells to Tau-induced neurodegeneration. While much attention has been focused on the toxic effects of abnormal Tau aggregates in neurons, emerging evidence suggests that Tau pathology can also impact glial cells, particularly astrocytes and microglia, leading to neuroinflammation and neuronal dysfunction. Tau aggregates can activate microglia, the resident immune cells of the central nervous system, leading to a pro-inflammatory response (Morales et al., 2013). Activated microglia release cytokines, chemokines, and reactive oxygen species, contributing to neuroinflammation and neuronal damage. Chronic activation of microglia by Tau pathology may exacerbate neurodegeneration and disease progression (Yoshiyama et al., 2007). Glial cells, particularly astrocytes, have been implicated in the propagation of Tau pathology. Recent studies suggest that astrocytes can internalize and spread pathological Tau aggregates, potentially contributing to the spreading of Tau pathology throughout the brain. Our findings of Tau toxicity in astrocytes and microglial cells, therefore, can be explained by the release of toxic Tau species by neurons in the extracellular compartment, and potentially by their internalization by glial cells. At the same time, the protective effects of SUMO2 overexpression may also originate from neuronal expression. In fact, we did not observe robust expression of SUMO2 directly in glial cells, nonetheless their dramatic rescue suggests that the neuronal expression of SUMO2 might trigger non-cell autonomous protective mechanisms in astrocytes and microglia.

Previous studies on cellular models of FTD demonstrated a hypertrophy of FTD astrocytes and increased levels of GFAP compared to control astrocytes (Hallmann et al., 2017). In contrast with those reports, our results of astrocyte atrophy instead agree with a work conducted in human FTD samples, where the authors also describe astrocyte atrophy (Rodríguez et al., 2024). The discrepancies between our findings and those by Hallmann et al., could derive the different experimental conditions, such as time of expression and amount of Tau expressed. Interestingly, similar atrophy of astrocytes was also reported in AD brains (Rodríguez et al., 2023). Moreover, our results also suggest that TauP301L induces both atrophy and degeneration of glial cells. This highlights the significance of our study using a viral approach to express only modest levels of human Tau carrying the FTD-associated mutation P301L samples. Our data in fact reproduce the findings obtained in human samples (Broe et al., 2004).

Overall, Tau toxicity toward glial cells represents an important aspect of Tauopathies, contributing to neuroinflammation, synaptic dysfunction, and neuronal damage. Understanding the mechanisms underlying Tau-mediated glial dysfunction may provide insights into the pathogenesis of Tauopathies and identify potential therapeutic targets for these devastating neurodegenerative diseases.

At the same time, while the effects of SUMOylation in the brain have been primarily investigated in neurons, there is evidence of dysregulation of SUMOylation pathways in astrocytes and microglia (Wong et al., 2013), which has been implicated in various neurological disorders, including neuroinflammation (Karhausen et al., 2018; Akar and Feinstein, 2009), neurodegeneration, and brain injury (Maruyama et al., 2018). Further research into the specific targets and mechanisms of SUMOylation in glial cells may offer new insights into the pathogenesis of these disorders and potential therapeutic strategies. More specifically, investigating the dynamic regulation of SUMOylation and its impact on AD-related proteins, such as Tau, may provide insights into the mechanisms underlying AD pathophysiology and potential therapeutic targets for the disease.

## Data Availability

The original contributions presented in the study are included in the article/Supplementary material, further inquiries can be directed to the corresponding author.

## Supplementary material

SUPPLEMENTARY FIGURE 1 Representative image of total Tau immunoblots from 11 weeks-old WT mice injected with PBS or AAV transducing TauP301L (1.3x10^9^ IU) for 17 weeks. The molecular weight corresponding to the 2N4R human Tau (68kDa) and the mouse Tau (50kDa) were well separated in order to quantify the percentage of human Tau over the total Tau. We found that that the human TauP301L protein was 10% of the total Tau expressed in the hippocampi after AAV transduction.

SUPPLEMENTARY FIGURE 2 Analysis of Tau phosphoresidues performed on western blots in mice injected with PBS, P301L-GFP AAV alone, or P301L-GFP AAV in combination with SUMO2-RFP AAV. Quantification of western blots for the indicated phosphoresidues, expressed as pTau over total Tau. One-way ANOVA followed by Tukey’s multiple comparisons test; **p*<0.05, ***p*<0,001, *n*=4,8,8 (A) and *n*=4,8,9 (B, C, D, E).

SUPPLEMENTARY FIGURE 3 Neuronal degeneration in pyramidal cells expressing hTauP301L. **(A)** Representative image of pyramidal neurons from mice that received hippocampal injections of AAV transducing 2N4R h TauP301L (1.3x10^9^ IU). TauP301L included an HA tag at the C terminus of Tau and was expressed together with GFP under the same promoter. HA immunostaining in magenta shows the TauP301L protein expression and nuclei (stained with DAPI) in blue. **(B)** Representative image and quantification of chromatolysis in hippocampal regions in Cresyl violet stained sections. Scale bar 10um. Axonal degeneration and condensed nuclei are evident in neurons expressing high levels of hTauP301L One-way ANOVA followed by Tukey’s test, ***p*<0.01, *n*= 7, 10, 8 (CA1+2) and *n*=7, 9, 8 (CA3).

## References

[ref1] Akar C. A. Feinstein D. L. (2009). Modulation of inducible nitric oxide synthase expression by sumoylation. J. Neuroinflammation 6:12. doi: 10.1186/1742-2094-6-12, PMID: 19323834 PMC2667488

[ref2] Anderson D. B. Zanella C. A. Henley J. M. Cimarosti H. (2017). Sumoylation: implications for neurodegenerative diseases. Adv. Exp. Med. Biol. 963, 261–281. doi: 10.1007/978-3-319-50044-7_16 28197918

[ref3] Bernstock J. D. Yang W. Ye D. G. Shen Y. Pluchino S. Lee Y.-J.. (2018). SUMOylation in brain ischemia: patterns, targets, and translational implications. J. Cereb. Blood Flow Metab. 38, 5–16. doi: 10.1177/0271678X17742260, PMID: 29148315 PMC5757445

[ref4] Bouchard D. Wang W. Yang W.-C. He S. Garcia A. Matunis M. J. (2021). SUMO paralogue–specific functions revealed through systematic analysis of human knockout cell lines and gene expression data. Mol. Biol. Cell 32, 1849–1866. doi: 10.1091/mbc.E21-01-0031, PMID: 34232706 PMC8684707

[ref5] Braak H. Braak E. (1995). Staging of Alzheimer’s disease-related neurofibrillary changes. Neurobiol. Aging 16, 271–278. doi: 10.1016/0197-4580(95)00021-6 7566337

[ref6] Broe M. Kril J. Halliday G. M. (2004). Astrocytic degeneration relates to the severity of disease in frontotemporal dementia. Brain 127, 2214–2220. doi: 10.1093/brain/awh250 15282215

[ref7] Celen A. B. Sahin U. (2020). Sumoylation on its 25th anniversary: mechanisms, pathology, and emerging concepts. FEBS J. 287, 3110–3140. doi: 10.1111/febs.15319, PMID: 32255256

[ref9] Chenfei Z. Haizhen Y. Jie X. Na Z. Bo X. (2022). Effects of aerobic exercise on hippocampal SUMOylation in APP/PS1 transgenic mice. Neurosci. Lett. 767:136303. doi: 10.1016/j.neulet.2021.136303, PMID: 34695453

[ref10] Cho S.-J. Yun S.-M. Jo C. Lee D.-H. Choi K. J. Song J. C.. (2015). SUMO1 promotes Aβ production via the modulation of autophagy. Autophagy 11, 100–112. doi: 10.4161/15548627.2014.984283, PMID: 25484073 PMC4502770

[ref11] Dorval V. Fraser P. E. (2006). Small ubiquitin-like modifier (SUMO) modification of natively unfolded proteins tau and α-Synuclein. J. Biol. Chem. 281, 9919–9924. doi: 10.1074/jbc.M510127200, PMID: 16464864

[ref12] Dorval V. Fraser P. E. (2007). SUMO on the road to neurodegeneration. Biochim. Biophys. Acta 1773, 694–706. doi: 10.1016/j.bbamcr.2007.03.017, PMID: 17475350

[ref13] Drisaldi B. Colnaghi L. Fioriti L. Rao N. Myers C. Snyder A. M.. (2015). SUMOylation is an inhibitory constraint that regulates the prion-like aggregation and activity of CPEB3. Cell Rep. 11, 1694–1702. doi: 10.1016/j.celrep.2015.04.061, PMID: 26074071 PMC5477225

[ref14] Enserink J. M. (2015). Sumo and the cellular stress response. Cell Div. 10:4. doi: 10.1186/s13008-015-0010-1, PMID: 26101541 PMC4476178

[ref15] Ficulle E. Shah Sufian M. D. Tinelli C. Corbo M. Feligioni M. (2018). Aging-related SUMOylation pattern in the cortex and blood plasma of wild type mice. Neurosci. Lett. 668, 48–54. doi: 10.1016/j.neulet.2018.01.004, PMID: 29325714

[ref16] Ford L. Fioriti L. Kandel E. R. (2020). Ubiquitination and SUMOylation of amyloid and amyloid-like proteins in health and disease. Curr. Issues Mol. Biol. 35, 195–230. doi: 10.21775/cimb.035.195, PMID: 31422940

[ref17] Hallmann A.-L. Araúzo-Bravo M. J. Mavrommatis L. Ehrlich M. Röpke A. Brockhaus J.. (2017). Astrocyte pathology in a human neural stem cell model of frontotemporal dementia caused by mutant TAU protein. Sci. Rep. 7:42991. doi: 10.1038/srep42991, PMID: 28256506 PMC5335603

[ref18] Hardy J. Higgins G. (1992). Alzheimer’s disease: the amyloid Cascade hypothesis. Science 256, 184–185. doi: 10.1126/science.1566067 1566067

[ref20] Jaworski T. Dewachter I. Lechat B. Croes S. Termont A. Demedts D.. (2009). AAV-tau mediates pyramidal neurodegeneration by cell-cycle re-entry without neurofibrillary tangle formation in wild-type mice. PLoS One 4:e7280. doi: 10.1371/journal.pone.0007280, PMID: 19794916 PMC2748684

[ref21] Karhausen J. Bernstock J. D. Johnson K. R. Sheng H. Ma Q. Shen Y.. (2018). Ubc9 overexpression and SUMO1 deficiency blunt inflammation after intestinal ischemia/reperfusion. Lab. Invest. 98, 799–813. doi: 10.1038/s41374-018-0035-6, PMID: 29472640 PMC6397426

[ref23] Knock E. Matsuzaki S. Takamura H. Satoh K. Rooke G. Han K.. (2018). SUMO1 impact on Alzheimer disease pathology in an amyloid-depositing mouse model. Neurobiol. Dis. 110, 154–165. doi: 10.1016/j.nbd.2017.11.015, PMID: 29217476 PMC9024178

[ref24] Koga S. Kojima A. Kuwabara S. Yoshiyama Y. (2014). Immunohistochemical analysis of tau phosphorylation and Astroglial activation with enhanced leptin receptor expression in diet-induced obesity mouse Hippocampus. Neurosci. Lett. 571, 11–16. doi: 10.1016/j.neulet.2014.04.028, PMID: 24785100

[ref25] Kovacs G. G. (2017). Tauopathies. Handb. Clin. Neurol. 145, 355–368. doi: 10.1016/B978-0-12-802395-2.00025-0 28987182

[ref26] Kusaka H. Imai T. Hashimoto S. Yamamoto T. Maya K. Yamasaki M. (1988). Ultrastructural study of chromatolytic neurons in an adult-onset sporadic case of amyotrophic lateral sclerosis. Acta Neuropathol. 75, 523–528. doi: 10.1007/BF00687142. PMID: 3376756, PMID: 3376756

[ref27] Lee L. Dale E. Staniszewski A. Zhang H. Saeed F. Sakurai M.. (2014). Regulation of synaptic plasticity and cognition by SUMO in Normal physiology and Alzheimer’s disease. Sci. Rep. 4:7190. doi: 10.1038/srep07190, PMID: 25448527 PMC4250909

[ref28] Lee Y.-j. Miyake S.-i. Wakita H. McMullen D. C. Azuma Y. Auh S.. (2006). Protein SUMOylation is massively increased in hibernation torpor and is critical for the cytoprotection provided by ischemic preconditioning and hypothermia in SHSY5Y cells. J. Cereb. Blood Flow 27, 950–962. doi: 10.1038/sj.jcbfm.9600395, PMID: 16955077 PMC2396349

[ref29] Lee Y.-J. Mou Y. Klimanis D. Bernstock J. D. Hallenbeck J. M. (2014). Global SUMOylation is a molecular mechanism underlying hypothermia-induced ischemic tolerance. Front. Cell. Neurosci. 8:416. doi: 10.3389/fncel.2014.00416, PMID: 25538566 PMC4255597

[ref30] Lee Y.-j. Mou Y. Maric D. Klimanis D. Auh S. Hallenbe J. M. (2011). Elevated global SUMOylation in Ubc9 transgenic mice protects their brains against focal cerebral ischemic damage. PLoS One 6:e25852. doi: 10.1371/journal.pone.0025852, PMID: 22016779 PMC3189225

[ref31] Levine S. Saltzman A. Kumar A. R. (2004). A method for peripheral chromatolysis in neurons of trigeminal and dorsal root ganglia, produced in rats by lithium. J. Neurosci. Methods 132, 1–7. doi: 10.1016/j.jneumeth.2003.07.001, PMID: 14687669

[ref32] Lewis J. McGowan E. Rockwood J. Melrose H. Nacharaju P. Van Slegtenhorst M.. (2000). Neurofibrillary tangles, Amyotrophy and progressive motor disturbance in mice expressing mutant (P301L) tau protein. Nat. Genet. 25, 402–405. doi: 10.1038/78078, PMID: 10932182

[ref33] Luo H.-B. Xia Y.-Y. Shu X.-J. Liu Z.-C. Feng Y. Liu X.-H.. (2014). SUMOylation at K340 inhibits tau degradation through deregulating its phosphorylation and ubiquitination. Proc. Natl. Acad. Sci. 111, 16586–16591. doi: 10.1073/pnas.1417548111, PMID: 25378699 PMC4246270

[ref34] Manza L. L. Codreanu S. G. Stamer S. L. Smith D. L. Sam Wells K. Roberts R. L.. (2004). Global shifts in protein Sumoylation in response to electrophile and oxidative stress. Chem. Res. Toxicol. 17, 1706–1715. doi: 10.1021/tx049767l, PMID: 15606148

[ref35] Maruyama T. Wada H. Abe Y. Niikura T. (2018). Alteration of global protein SUMOylation in neurons and astrocytes in response to Alzheimer’s disease-associated insults. Biochem. Biophys. Res. Commun. 500, 470–475. doi: 10.1016/j.bbrc.2018.04.104, PMID: 29660340

[ref36] Matsuzaki S. Lee L. Knock E. Srikumar T. Sakurai M. Hazrati L.-N.. (2015). SUMO1 affects synaptic function, spine density and memory. Sci. Rep. 5:10730. doi: 10.1038/srep10730, PMID: 26022678 PMC4650663

[ref37] McIlwain D. L. Hoke V. B. (2005). The role of the cytoskeleton in cell body enlargement, increased nuclear eccentricity and chromatolysis in axotomized spinal motor neurons. BMC Neurosci. 6:19. doi: 10.1186/1471-2202-6-19 15774011 PMC1079867

[ref38] McMillan L. E. Brown J. T. Henley J. M. Cimarosti H. (2011). Profiles of SUMO and ubiquitin conjugation in an Alzheimer’s disease model. Neurosci. Lett. 502, 201–208. doi: 10.1016/j.neulet.2011.07.045, PMID: 21843595 PMC3176896

[ref39] Mendler L. Braun T. Müller S. (2016). The ubiquitin-like SUMO system and heart function. Circ. Res. 118, 132–144. doi: 10.1161/CIRCRESAHA.115.307730, PMID: 26837744

[ref40] Morales I. Jiménez J. M. Mancilla M. Maccioni R. B. (2013). Tau oligomers and fibrils induce activation of microglial cells. J. Alzheimer’s Dis. 37, 849–856. doi: 10.3233/JAD-131843, PMID: 23948931

[ref41] Nilson A. N. English K. C. Gerson J. E. Barton Whittle T. Nicolas Crain C. Xue J.. (2017). Tau oligomers associate with inflammation in the brain and retina of Tauopathy mice and in neurodegenerative diseases. J. Alzheimer’s Dis. 55, 1083–1099. doi: 10.3233/JAD-160912, PMID: 27716675 PMC5147514

[ref42] Nisticò R. Ferraina C. Marconi V. Blandini F. Negri L. Egebjerg J.. (2014). Age-related changes of protein SUMOylation balance in the AβPP Tg2576 mouse model of Alzheimer’s disease. Exper. Pharmacol. Drug Discov. 5:63. doi: 10.3389/fphar.2014.00063, PMID: 24778618 PMC3985012

[ref43] Orsini F. Argyrousi E. Restelli E. Ford L. K. Takamura H. Matsuzaki S.. (2022). SUMO2 protects against tau-induced synaptic and cognitive dysfunction. bioRxiv. doi: 10.1101/2022.11.11.516192

[ref44] Orsini F. Chrysanthou E. Thomas Dudler W. Cummings J. Takahashi M. Fujita T.. (2016). Mannan binding lectin-associated serine Protease-2 (MASP-2) critically contributes to post-ischemic brain injury independent of MASP-1. J. Neuroinflammation 13:213. doi: 10.1186/s12974-016-0684-6, PMID: 27577570 PMC5006610

[ref45] Orsini F. Villa P. Parrella S. Zangari R. Zanier E. R. Gesuete R.. (2012). Targeting mannose-binding lectin confers long-lasting protection with a surprisingly wide therapeutic window in cerebral ischemia. Circulation 126, 1484–1494. doi: 10.1161/CIRCULATIONAHA.112.103051, PMID: 22879370 PMC3478764

[ref46] Patel H. Dobson R. J. B. Newhouse S. J. (2019). A Meta-Analysis of Alzheimer’s Disease Brain Transcriptomic Data. J. Alzheimers Dis. 68, 1635–1656. doi: 10.3233/JAD-181085 30909231 PMC6484273

[ref47] Reid M. J. Beltran-Lobo P. Johnson L. Perez-Nievas B. G. Noble W. (2020). Astrocytes in Tauopathies. Front. Neurol. 11:572850. doi: 10.3389/fneur.2020.572850, PMID: 33071951 PMC7542303

[ref48] Rodríguez J. J. Zallo F. Gardenal E. Cabot J. Busquets X. (2023). Prominent and conspicuous astrocyte atrophy in human sporadic and familial Alzheimer’s disease. Brain Struct. Funct. 228, 2103–2113. doi: 10.1007/s00429-023-02707-x, PMID: 37730895 PMC10587264

[ref49] Rodríguez J. J. Zallo F. Gardenal E. Cabot J. Busquets X. (2024). Entorhinal cortex astrocytic atrophy in human frontotemporal dementia. Brain Struct. Funct. 229, 695–703. doi: 10.1007/s00429-024-02763-x, PMID: 38308043

[ref50] Ryu H.-Y. Ahn S. H. Hochstrasser M. (2020). SUMO and cellular adaptive mechanisms. Exp. Mol. Med. 52, 931–939. doi: 10.1038/s12276-020-0457-2, PMID: 32591648 PMC7338444

[ref51] Sang J. Yang K. Sun Y. Han Y. Cang H. Chen Y.. (2011). SUMO2 and SUMO3 transcription is differentially regulated by oxidative stress in an Sp1-dependent manner. Biochem. J. 435, 489–498. doi: 10.1042/BJ20101474, PMID: 21291420

[ref52] Sasaki A. Kawarabayashi T. Murakami T. Matsubara E. Ikeda M. Hagiwara H.. (2008). Microglial activation in brain lesions with tau deposits: Comparison of human Tauopathies and tau transgenic mice TgTauP301L. Brain Res. 1214, 159–168. doi: 10.1016/j.brainres.2008.02.084 18457819

[ref53] Suk T. R. Nguyen T. T. Fisk Z. A. Mitkovski M. Geertsma H. M. Parmasad J.-L. A.. (2023). Characterizing the differential distribution and targets of Sumo1 and Sumo2 in the mouse brain. IScience 26:106350. doi: 10.1016/j.isci.2023.106350, PMID: 37009224 PMC10060683

[ref54] Vertegaal A. C. O. (2022). Signalling mechanisms and cellular functions of SUMO. Nat. Rev. Mol. Cell Biol. 23, 715–731. doi: 10.1038/s41580-022-00500-y 35750927

[ref55] Wang W. Matunis M. J. (2024). Paralogue-specific roles of SUMO1 and SUMO2/3 in protein quality control and associated diseases. Cells 13:8. doi: 10.3390/cells13010008, PMID: 38201212 PMC10778024

[ref56] Wong M. B. Goodwin J. Norazit A. Meedeniya A. C. B. Richter-Landsberg C. Gai W. P.. (2013). SUMO-1 is associated with a subset of lysosomes in glial protein aggregate diseases. Neurotox. Res. 23, 1–21. doi: 10.1007/s12640-012-9358-z, PMID: 23229893

[ref57] Yang W. Huaxin Sheng H. Homi M. Warner D. S. Paschen W. (2008). Cerebral Ischemia/Stroke and Small Ubiquitin-like Modifier (SUMO) Conjugation--a New Target for Therapeutic Intervention? J. Neurochem. 106, 989–999. doi: 10.1111/j.1471-4159.2008.05404.x 18410505

[ref58] Yang W. Paschen W. (2015). SUMO proteomics to decipher the SUMO-modified proteome regulated by various diseases. Proteomics 15, 1181–1191. doi: 10.1002/pmic.201400298, PMID: 25236368 PMC4382800

[ref59] Yang Y. Xia Z. Wang X. Zhao X. Sheng Z. Yang Ye G.. (2018). Small-molecule inhibitors targeting protein SUMOylation as novel anticancer compounds. Mol. Pharmacol. 94, 885–894. doi: 10.1124/mol.118.112300, PMID: 29784649

[ref60] Yoshiyama Y. Higuchi M. Zhang B. Huang S.-M. Iwata N. Saido T. C.. (2007). Synapse loss and microglial activation precede tangles in a P301S Tauopathy mouse model. Neuron 53, 337–351. doi: 10.1016/j.neuron.2007.01.010, PMID: 17270732

