# Supplementary figures and images for: SUMO2 rescues neuronal and glial cells from the toxicity of P301L Tau mutant
Source: Front Cell Neurosci. 2024 Dec 12;18:1437995. doi: 10.3389/fncel.2024.1437995 (PMC11669524; doi:10.3389/fncel.2024.1437995)

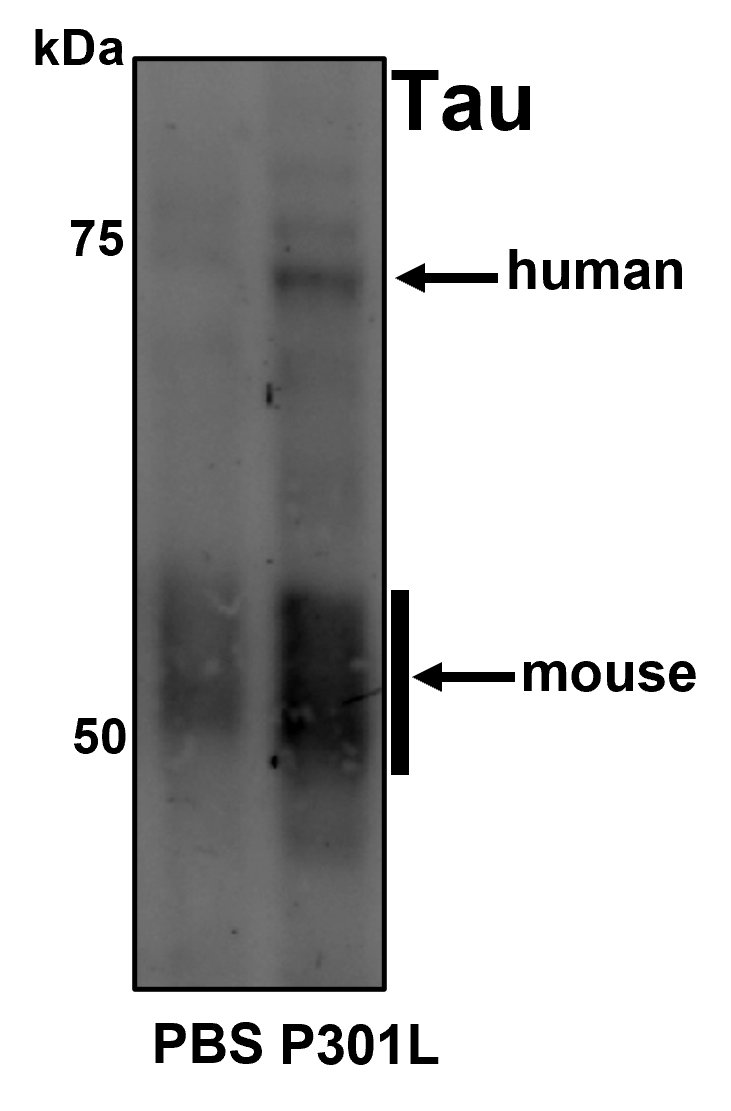

Supplement: SUPPLEMENTARY FIGURE 1 — Representative image of total Tau immunoblots from 11 weeks-old WT mice injected with PBS or AAV transducing TauP301L (1.3x10^9^ IU) for 17 weeks. The molecular weight corresponding to the 2N4R human Tau (68kDa) and the mouse Tau (50kDa) were well separated in order to quantify the percentage of human Tau over the total Tau. We found that that the human TauP301L protein was 10% of the total Tau expressed in the hippocampi after AAV transduction. [file Image_1.TIF]

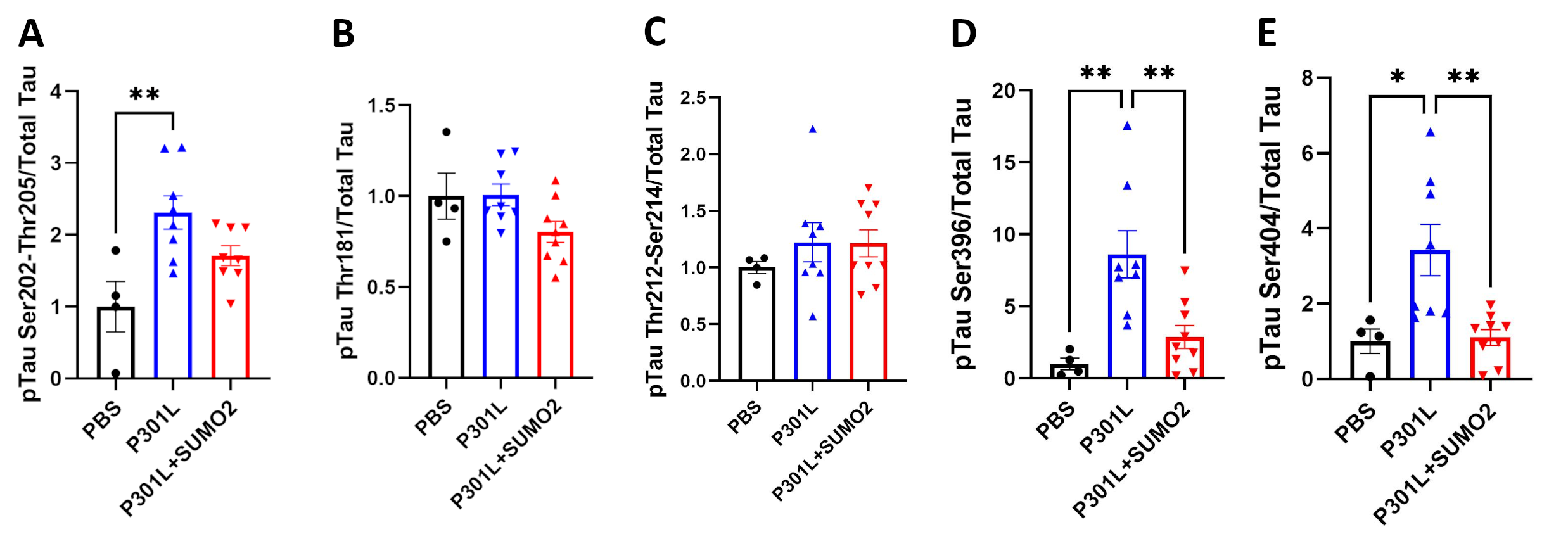

Supplement: SUPPLEMENTARY FIGURE 2 — Analysis of Tau phosphoresidues performed on western blots in mice injected with PBS, P301L-GFP AAV alone, or P301L-GFP AAV in combination with SUMO2-RFP AAV. Quantification of western blots for the indicated phosphoresidues, expressed as pTau over total Tau. One-way ANOVA followed by Tukey’s multiple comparisons test; *p<0.05, **p<0,001, n=4,8,8 (A) and n=4,8,9 (B, C, D, E). [file Image_2.TIFF]

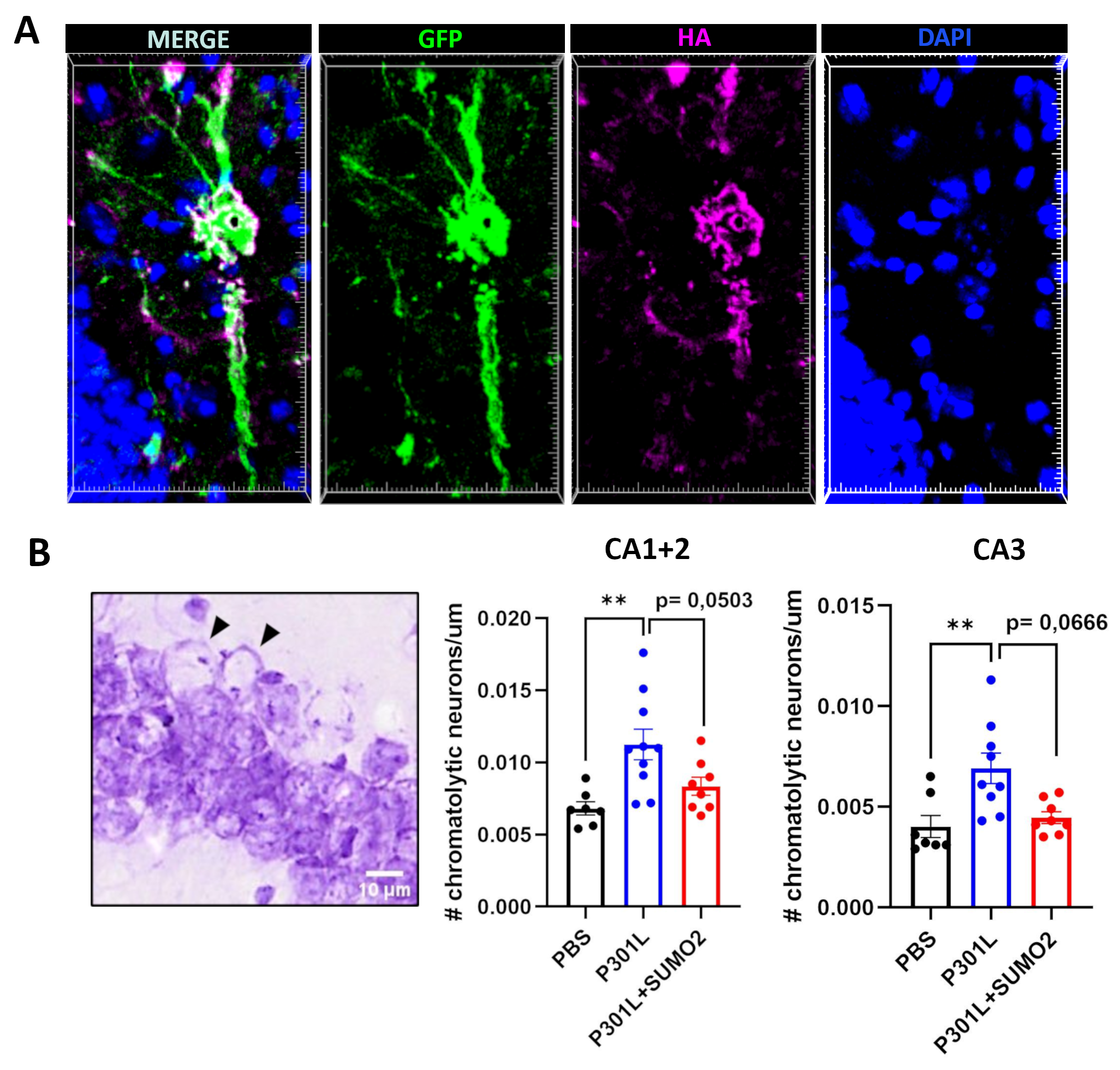

Supplement: SUPPLEMENTARY FIGURE 3 — Neuronal degeneration in pyramidal cells expressing hTauP301L. (A) Representative image of pyramidal neurons from mice that received hippocampal injections of AAV transducing 2N4R h TauP301L (1.3x10^9^ IU). TauP301L included an HA tag at the C terminus of Tau and was expressed together with GFP under the same promoter. HA immunostaining in magenta shows the TauP301L protein expression and nuclei (stained with DAPI) in blue. (B) Representative image and quantification of chromatolysis in hippocampal regions in Cresyl violet stained sections. Scale bar 10um. Axonal degeneration and condensed nuclei are evident in neurons expressing high levels of hTauP301L One-way ANOVA followed by Tukey’s test, **p<0.01, n= 7, 10, 8 (CA1+2) and n=7, 9, 8 (CA3). [file Image_3.TIFF]
